# Mechanism of Interlaminar Strengthening and Toughening of Al/CFRP/Al Composite Laminates

**DOI:** 10.3390/ma16020560

**Published:** 2023-01-06

**Authors:** Jian Wang, Tianqi Qiao, Aidong Wang, Xiudong Li, Tao Wang

**Affiliations:** 1 National Engineering Research Center for Equipment and Technology of Cold Rolled Strip, Yanshan University, Qinhuangdao 066004, China; 2 Engineering Research Center of Advanced Metal Composites Forming Technology and Equipment, Ministry of Education, Taiyuan University of Technology, Taiyuan 030024, China

**Keywords:** Al/CFRP/Al, carbon nanotubes, strength and toughness, shear strength, impact resistance

## Abstract

To solve the problem of easy delamination of the interface of Al/CFRP/Al laminate (CFRP, carbon fiber composite; Al, aluminum), a method of constructing high strength and toughness at the interface is proposed. The effects of adding resin and carbon nanotubes at the interface on the shear, flexural strength and impact resistance of Al/CFRP/Al laminates are studied. Experimental results show that gullies and micropores can be formed on the surface of aluminum after pretreatment, and the contact area between aluminum and matrix can be increased. After resin is added to the Al/CFRP interface, the shear strength between layers and the bending strength and impact resistance of laminates are improved. Mixing carbon nanotubes into the added resin will further improve the mechanical properties of laminates. On the basis of strengthening and toughening the Al/CFRP interface, the carbon nanotubes added between the CFRP layers can strengthen and toughen the resin at the interface, which enhances the bending strength and impact resistance of the laminates. When the resin between the layers breaks, the carbon nanotubes will be pulled off and pulled out to resist the damage of the resin, and the carbon nanotubes at the end of the crack can hinder the propagation of the cracks.

## 1. Introduction

Fiber metal laminates are composed of metal laminates and fiber-reinforced composite materials. The laminates combine the advantages of metal toughness and fiber composites with high strength, high stiffness, and light weight [1,2]. They are widely used in aerospace, industry, automobiles, ships, and other fields. Fiber metal laminates are formed by stacking two materials, and the interface between adjacent layers is the weakest in the laminate, which is prone to delamination. The interlayer interface needs better properties to maximize the performance of fiber metal laminates. The interface performance greatly affects the mechanical and physical properties of laminates [3,4,5,6,7]. The interface between metal and matrix influences the combination and stress transfer effect of metal and fiber layers, and it determines the failure behavior and comprehensive properties of fiber metal laminates [8]. The bonding method between fiber metal laminates is mainly adhesive bonding [9]. The surface treatment of metal before bonding can improve the roughness of the metal surface first and then improve the bonding strength between metal and resin. Many scholars have used different methods to deal with the interfaces between composites for enhancing the interfacial strength of laminates. Xie et al. [10] provided an idea for interface bonding, called al-coating. Hamill et al. [11] treated the surface of aluminum plate by chemical and physical methods, and the interlayer performance of fiber metal laminates was improved after surface treatment. Hara et al. [12] added adhesive between fiber metal laminates and determined that the thickness of adhesive would affect the structural characteristics of fiber metal laminates. Xin et al. [13] treated titanium plates with annealing, sandblasting and anodizing, and they studied the effect of interface strength on the mechanical properties of Ti/CFRP/Ti laminates through indentation test and low-speed impact test. The results show that after sandblasting and anodizing on the metal surface, the interface strength is greatly improved, and then the performance of FMLs is improved. Zhuwei et al. [14] prepared Al/CFRP/Al laminate after surface treatment of A6061. The results show that the interface strength of Al/CFRP after surface treatment is improved, and the shear strength of Al/CFRP/Al laminate modified by a silane coupling agent and pretreated by phosphoric acid anodizing is the best.

Carbon nanotubes have excellent mechanical properties. When a certain amount of carbon nanotubes are added to the resin, the bending strength, tensile strength, and tensile elastic modulus of the material are greatly improved [15,16,17,18,19,20]. Carbon nanotubes can give full play to their role in bearing, bridging and preventing crack propagation. Hong et al. [21] added carbon nanotube films between fiber resin layers and measured the bending properties and interlaminar shear properties of the materials. The results show that the bending properties of the composites are 16.04% higher than those of the composites without the addition. Adding an appropriate amount of carbon nanotubes can form a network structure in the matrix, hinder crack propagation, and improve the mechanical properties of the material. Zhang et al. [22] dispersed multi-walled carbon nanotubes (MWCNTs) in polyimide and filled them between the metal and composite. This method improves the interfacial properties of the laminate. In this paper, the effect of interfacial treatment on interlaminar shear strength, flexural strength, and impact resistance was analyzed by the surface treatment of aluminum plate and the addition of resin and carbon nanotubes between Al/CFRP/Al laminates. The method of constructing high strength and toughness at the interface is proposed to solve the problem of easy delamination of this type of composite in the service process. For example, when the laminates receive various impacts, the invisible delamination in the laminates will affect the service performance. The laminates with carbon nanotubes have good interlaminar strength, and it is difficult to delaminate when receiving various external loads, which will improve the service performance of the laminates.

## 2. Materials and Methods

### 2.1. Materials

The type of aluminum plate used in this experiment is AA6061. The T700 unidirectional carbon fiber prepreg and added epoxy resin used are provided by Zhongfu Shenying Carbon Fiber Co., Ltd. (Lianyungang, China), and the thickness of the prepreg is 0.1 mm. MWCNTs are provided by Nanjing Xianfeng Nano Co., Ltd. (Nanjing, China), with a purity of 99% and a diameter of about 30 nm. Nitric acid and sodium hydroxide reagents are provided by Kaitong (Tianjin, China).

### 2.2. Preparation of Laminates

Four laminates with different structures are prepared, as shown in Table 1, where “e” indicates that epoxy resin is added between aluminum and CFRP, and “ec” indicates that epoxy resin mixed with carbon nanotubes is added between aluminum and CFRP; “CFRPc“ refers to CFRP with carbon nanotubes mixed between layers.

**Figure 1:**
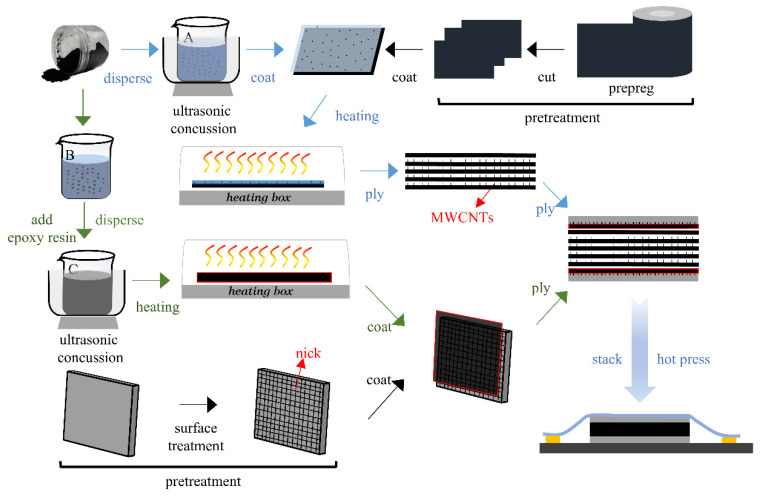
Schematic of laminate preparation process.

Figure 1 shows the manufacturing process of the whole laminate. Pretreatment includes cutting the prepreg and surface treatment of the aluminum plate. Surface treatment specifically refers to grinding, nicking and acid–alkali washing of the aluminum plate surface. The aluminum plate is sanded with 80-grit sandpaper first. Then, the knife is used to make horizontal and vertical nicks. The distance between adjacent nicks is required to be 5 mm, and the force is balanced in the process of nicking. Acid–alkali washing refers to soaking the aluminum plate after nicking in 100 g/L NaOH solution and 100 g/L nitric acid solution for 3 min and rinsing with water. The surface treatment increases the contact area between the aluminum plate and the resin matrix and forms a mechanical interlocking structure at the interface. At the same time, the prepregs are cut according to the experimental requirements. Laminate A directly stacks the two pretreated materials to form a sandwich structure. The Al/CFRP interface of laminate B is coated with epoxy resin and curing agent, and the mass ratio of curing agent to epoxy resin is 3:10.

Then, the carbon nanotube solution is prepared. Solution A is a carbon nanotube/ethanol solution. An appropriate volume of anhydrous ethanol and carbon nanotubes is mixed into a beaker to prepare a 1 wt% concentration of carbon nanotubes/ethanol solution. The beaker containing the carbon nanotube/ethanol solution is placed in an ultrasonic cleaning apparatus. After ultrasonic vibration for 1 h, a carbon nanotube/ethanol solution with uniformly dispersed carbon nanotubes is obtained. The configuration of solution B is as follows: A suitable volume of tetrahydrofuran and carbon nanotubes is mixed into a beaker to configure the carbon nanotube/tetrahydrofuran solution. Thereafter, a certain quantity of epoxy resin and curing agent is weighed and placed into the carbon nanotube/tetrahydrofuran solution. The mass ratio of carbon nanotube to epoxy resin is 1:100, and the mass ratio of curing agent to epoxy resin is 3:10. Then the solution is stirred until the epoxy resin is completely dissolved to form solution C. Similarly, the beaker containing solution C is placed into the ultrasonic cleaner for ultrasonic vibration for 1 h. Next, solution C is placed into the mold and baked in a heating box at 30 °C to obtain epoxy resin mixed with carbon nanotubes. This epoxy resin is coated on the interface between the aluminum plate and CFRP to form laminate C.

One side of the carbon fiber composite prepreg is uniformly coated with carbon nanotube/ethanol solution, and then it is placed in a heating box at about 30 °C for baking. After drying, the prepregs are stacked, and then the stacked prepregs are compounded with an aluminum plate coated with epoxy resin mixed with carbon nanotubes to obtain laminate D. Finally, the four laminates are hot pressed and cured.

### 2.3. Hot Pressing Scheme

**Figure 2:**
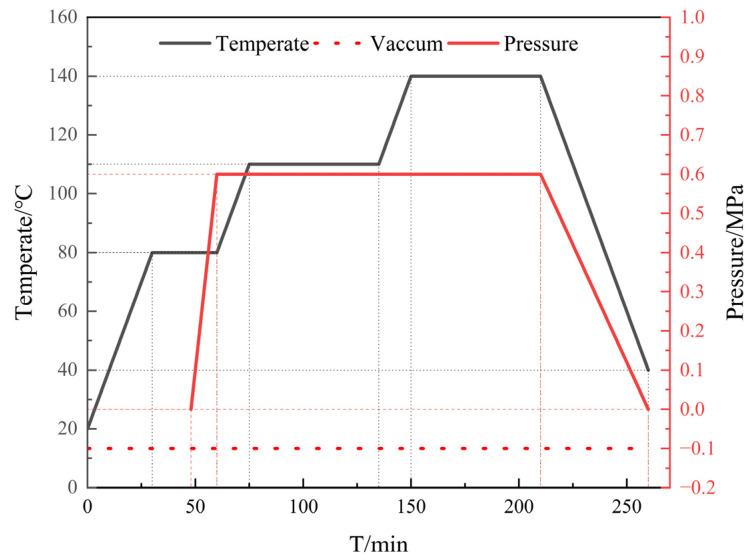
Laminate temperature and pressure curve.

The four kinds of laminates are placed in an autoclave (produced by Zhejiang puma Co., Ltd.(Zhejiang, China)) for hot press curing. The hot pressing scheme adopts three-stage curing to reduce the residual thermal stress of the laminate, and the curing process is shown in Figure 2. The temperature in the tank is first raised from room temperature to the pre-curing temperature (80 °C) of the resin at a heating rate of 2 °C/min, and the temperature is kept for 0.5 h to make the resin flow sufficiently and uniformly to ensure that the thermosetting resin thoroughly infiltrates the metal and removes air bubbles. Then, the tank is pressurized to 0.6 MPa at a pressurizing rate of 0.05 MPa/min by an air compressor and heated to 110 °C at the same heating rate and kept for 1 h. As a result, the resin in contact with the aluminum plate is first cured. Subsequently, the sample is heated to the post-curing temperature (140 °C) at the same heating rate, kept for 1 h until the resin is completely cured, and cooled to room temperature at a rate of 2 °C/min. The curing process is then ended.

### 2.4. Shearing Experiment

**Figure 3:**
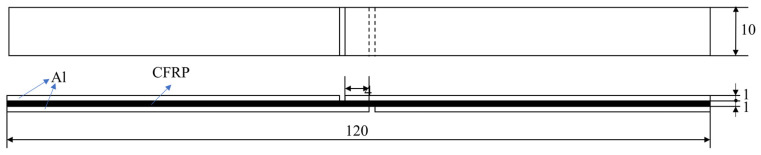
Dimensions of the sheared sample(mm).

Shear specimens are prepared in accordance with GB/T7124-2008 and subjected to shear testing on an Inspekt Table 100 kN tensile testing machine (Hegewald&Peschke, Dresden, Germany). The unidirectionally layered laminate is cut parallel to the fiber direction by a water jet to form a test piece with a length of 120 mm, a width of 10 mm, and a thickness of 3 mm. Here, the thickness of the upper and lower aluminum plates is 1 mm, and the specific size and structure are shown in Figure 3. Before the experiment, a gap is opened in the middle of the specimen, and the length of the shear plane is reserved to be 4 mm. The loading speed for the shear test is set to 1 mm/min, and three samples are tested for each condition.

The formula for shear strength is

(1) $$\emptyset =\frac{F_{max}}{b\times l}$$

where ∅ is the shear strength of the plane (MPa), $F_{max}$ is the maximum pull force (N), *b* is the width of the sample (mm), and *l* is the length of the sample (mm).

### 2.5. Bending Test

**Figure 4:**
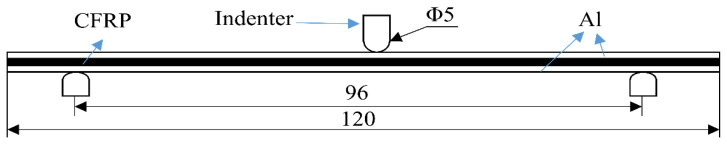
Dimensions of the bending specimen(mm).

The three-point bending test is performed following ASTMD7264. Similarly, three-point bending experiments are performed on an Inspekt Table 100 kN tensile testing machine. The unidirectional layered laminate is cut along the direction perpendicular to the fibers to form test pieces by water jet. The dimensions of the sample are 120 mm in length, 6 mm in width, and 3 mm in thickness. The span of the side support beam is 96 mm, and the loading speed is set to 5 mm/min. The specific size and structure are shown in Figure 4. The sample is aligned and centered on the support beam with its longitudinal axis perpendicular to the loading nose and side supports. The spacing between the support beams is 96 mm, and three samples are tested for each condition. The load is terminated when the maximum load value drops by 30% or the specimen fails.

The formula for three-point bending strength is

(2) $$\sigma =\frac{3PL}{{4bh}^{2}}$$

where *σ* is the outer surface stress of the laminate (MPa), *P* is the load force (N), *L* is the span width (mm), *b* is the width of the specimen (mm), and *h* is the thickness of the sample (mm).

### 2.6. Impact Test

The punch size is 20 mm and the mass is 21 kg. The size of the impact piece is set to 100 × 100 mm. The thickness of the aluminum plate is 0.6 mm, the core CFRP layup is [0°/90°]_2s_, and the total thickness of the laminate is 2 mm. The prepared specimen is placed on the impact platform, and the impact energy is set through the software. The system controls the rise of the punch to obtain potential energy, and free-fall impacts the specimen. After the impact contact, the punch rebounds, and the pneumatic device of the equipment controls the punch to prevent the influence of the secondary collision on the experimental results. The curve of the impact energy, load, displacement, and velocity with time during the impact process is obtained through the computer.

### 2.7. Metallographic Test

For the test piece requiring microscopic observation, cut the test piece into long strips with a water knife, then clamp one end of the strip on a vise, and cut off important parts with a saw blade. After that, put the cut test piece in the mold, add inlaid liquid cooling inlay to the mold, and take it out after standing for 6 h. Then polish the inlays with 400-, 1000- and 2000-mesh sandpaper in turn. During the polishing process, clean them with water from time to time to keep the polished surface wet, so as to prevent damage to the polished surface caused by incorrect polishing. Then grind with grinding paste on the MP-2C polishing machine. The rotation speed of the polishing machine is 2000 r/min, until the specimen can show a clear interface in the microscope without scratches. Then put it into the ZEISS Scope A1 metallographic microscope and observe the interface characteristics under the ZEISS Sigma 500 microscope (Oberkochen, Germany).

## 3. Results and Discussion

### 3.1. Laminate Manufacturing Effect

**Figure 5:**
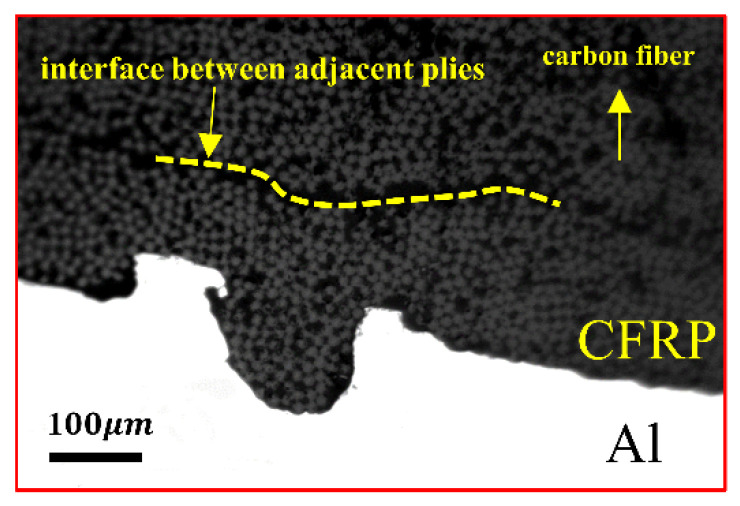
Microstructure of laminate A intercalation and nick.

Figure 5 shows the metallographic diagram of the CFRP/Al interface of laminate A. As shown in the figure, nothing is added to the CFRP/Al interface, and the matrix and fibers in the carbon fiber composite material are filled in the nicks. Carbon fibers enter the nick, and the interface of the layup is convex. This condition causes the distribution of fibers in the layup to change, and the fibers in the same layup are affected when transferring the load. This phenomenon of uneven distribution of fibers also exists in some micropores generated by acid–alkali washing, which affects the overall performance of the laminate.

**Figure 6:**
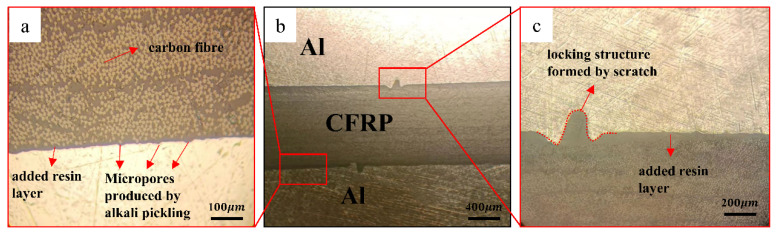
Microstructure of laminate B intercalation and nick.

Figure 6 shows the metallographic diagram of the CFRP/Al interface of laminate B. Figure 6b shows the structural diagram of the fiber metal laminate. The laminate is a sandwich structure: the upper and lower layers are aluminum plates, and the middle is carbon fiber composite. Figure 6c shows the interface interlocking structure. When the Al/CFRP/Al laminate is hot pressed and cured, the added epoxy resin enters the nick and closely combines with the aluminum plate to form a “W” interlocking structure, and no downward convex phenomenon occurs at the core CFRP/ply interface. Figure 6a shows the metallographic diagram of the higher magnification section. A layer of epoxy resin is added between the Al/CFRP interface to connect the two materials together. Micropores are generated on the surface of the aluminum plate after grinding and acid–alkali washing. Thus, the aluminum plate is closely combined with the resin to form a smaller interlocking structure, which improves the shear strength of the interface.

**Figure 7:**
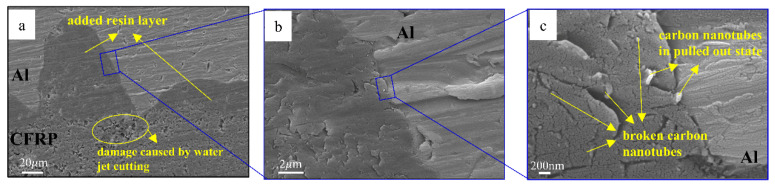
Interlayer carbon nanotube metallographic diagram at different magnification of laminate C.

Figure 7 shows the metallographic distribution of carbon nanotubes at the interface. The magnification of Figure 7a,b,c increases in turn. As shown in Figure 7a,b, the added resin is closely bonded with the aluminum layer. The accuracy of the water jet cutter is low and the resin will be damaged. Figure 7c shows the state of carbon nanotubes in the added epoxy resin. Most of the carbon nanotubes are inserted in the resin and are located at the end of large or small cracks. This finding shows that the existence of carbon nanotubes plays a role in preventing crack propagation. Some carbon nanotubes are also in the state of pulling out, and the larger pullout head is caused by a small amount of resin and gold spraying.

### 3.2. Shear Test Results

**Figure 8:**
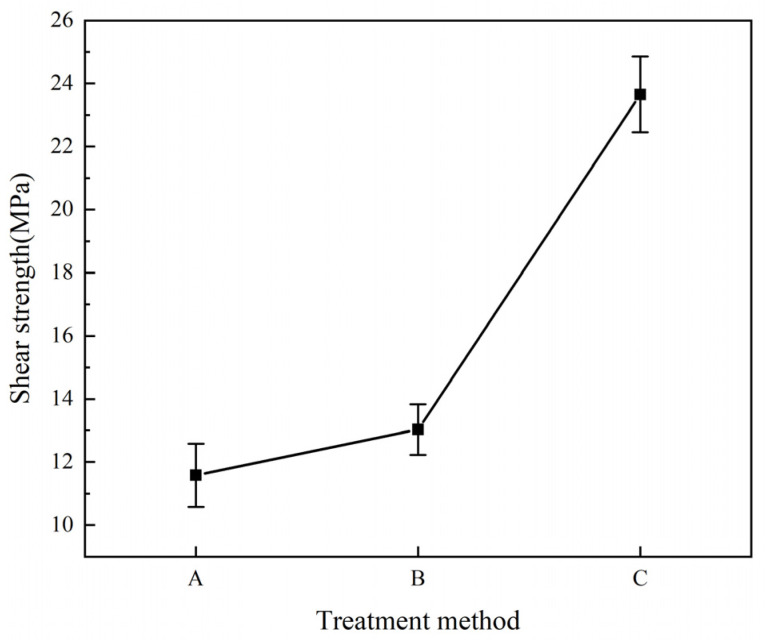
Shear strength curve of laminates after different treatment methods.

Figure 8 shows the shear strength values between the Al/CFRP interface of the three laminates after different interfacial treatments. Comparing the shear strength values of the three laminates indicates that adding resin between the Al/CFRP interface can improve the interlaminar shear strength, and adding carbon nanotubes in the resin can further improve the shear strength. Laminate C improves about 104% over laminate A. The shear strength reflects the composite strength of the two materials. The composite of the two materials is to give full play to the advantages of the two materials. The overall performance of the laminate is better when the composite strength is higher. If the composite strength is low, then the composite effect of the two materials is poor. The two materials are also easy to separate from each other, which will lose the significance of the composite plate and affect the performance of the laminate.

**Figure 9:**
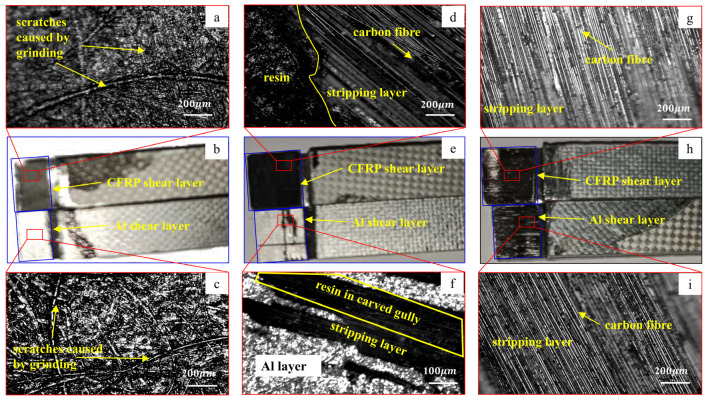
Shear failure morphologies of Al/CFRP bonding surfaces after different interface treatments.

**Figure 10:**
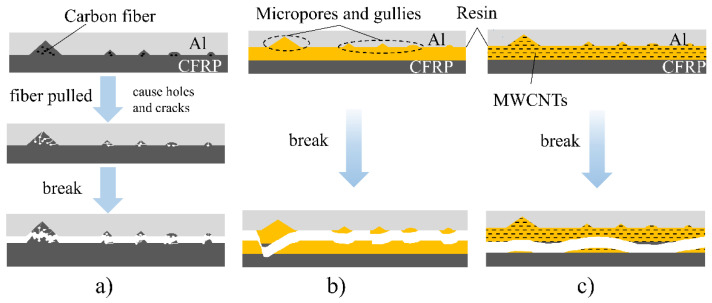
Schematic of shear failure between Al/CFRP layers after different interface treatments.

Figure 9 shows the failure of the joint surface of the two materials after the shear test of the three laminates. Figure 9a–c show the failure surface of laminate A. As shown from the figures, the joint surface of the CFRP layer and the aluminum plate is clean. It only shows tiny scratches caused by grinding, no resin can be observed on the surface of the aluminum plate, and the composite effect of the two materials is poor. Figure 10a is a schematic of the failure of laminate A. As shown in Figure 5, the matrix in the CFRP and the fibers enter the grooves and micropores of the aluminum plate during molding. The fiber elongation is small, and the grooves and pores cannot contain the entire fiber. Therefore, when the shearing experiment is performed on laminate A, when the laminates are subjected to external load and the interlayer is sheared, the micropores and grooves on the aluminum will pull out the fibers in the resin under the action of external force. Therefore, the resin in the microcracks will crack and the performance of the entire interface will be affected and damaged.

Figure 9d–f show the damaged morphology of the bonding surface of laminate B. As shown in the figures, some resin remains on the surface of the aluminum plate, while the resin and carbon fibers are exposed on the CFRP bonding surface. This result shows that after adding resin at the interface, the cohesion failure inside the resin and the peeling failure between the added resin and the aluminum plate occur at the CFRP/Al interface. Figure 9d shows that some carbon fibers are exposed, which indicates that damage has also occurred between the matrix resin and the added resin. Compared with the direct peeling of the two materials in laminate A, the diversity of the interlaminar failure of laminate B leads to improvement in the interlaminar shear strength. This finding also shows that the interface strength between the added epoxy resin and the aluminum plate is greater than the interface strength between the resin matrix and the aluminum plate, and the interlayer damage is transformed from the peeling of the aluminum plate and the resin to the damage inside the resin. Figure 10b shows the process of the destruction of laminate B, and the added resin flows into the holes uniformly. When the laminate is damaged by shearing, the interface between the aluminum plate and the added resin is peeled off, the interior of the resin is damaged, and damage between the added resin and the CFRP matrix resin also occurs.

Figure 9g–i are the failure surfaces of laminate C. As shown in the figures, after the epoxy resin mixed with carbon nanotubes is added, cohesive failure occurs between the layers of laminate C. The exposed carbon fiber on the damaged surface indicates that the damage also occurs between the two resins. This is the main failure mode of the Al/CFRP interface in laminate C. The interface treatment method greatly improves the interlaminar shear strength. Figure 10c shows that the resin mixed with carbon nanotubes is filled into the grooves generated by nicking, grinding, and acid–alkali washing, and the interlaminar damage becomes the damage between the added resin and the CFRP matrix.

**Figure 11:**
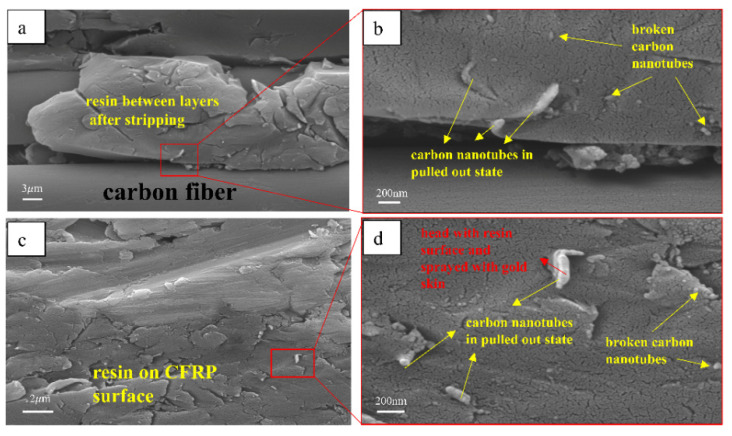
Metallographic diagram of shear failure of laminate C. (**a**) the electron microscope picture of the surface of the CFRP layer after shear failure of laminate C. (**b**) an enlarged view of (**a**). (**c**) the electron microscope picture of the surface of the CFRP layer after shear failure of laminate D. (**d**) an enlarged view of (**c**).

Figure 11 is the metallographic diagram of shear failure of laminate C. Figure 11a is the electron microscope picture of the surface of the CFRP layer after shear failure of laminate C. Cohesive failure occurs at the Al/CFRP interface, which results in some resin remaining on the surface of the CFRP layer and carbon nanotubes remaining on the surface of the residual resin. Figure 11b is an enlarged view of this part of the resin, and the carbon nanotubes show two states of fracture and pullout. Many cracks of different sizes are observed on the surface of the resin. Some carbon nanotubes are distributed at the end of the cracks, which can block the propagation of the crack. Carbon nanotubes resist the damage of load on the interface by pulling out and breaking. Figure 11c,d show the morphology of the resin and carbon nanotubes on the surface of the laminate after the shear experiment between the CFRP layers of laminate D. Among them, carbon nanotubes also show two states of pulling out and breaking. When laminate D is cured, the CFRP matrix resin flows and wraps the carbon nanotubes between the layers, and the strength of the interlayer resin is improved.

### 3.3. Bending Test Results

**Figure 12:**
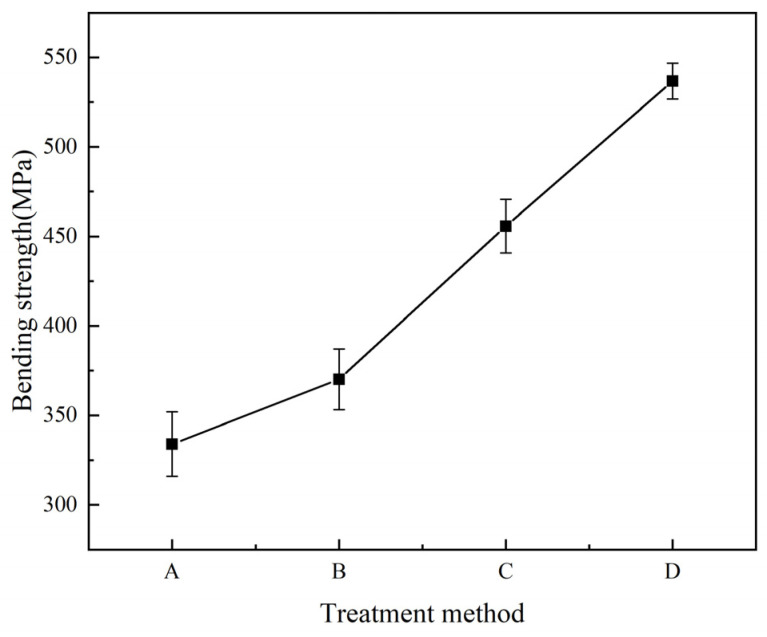
Bending strength curves of four laminates.

Figure 12 shows the bending strength of four kinds of laminates. As shown in the figure, the bending strength of the laminate is improved after resin is added to the Al/CFRP interface. The bending properties of laminates can be further improved by adding carbon nanotubes to the resin. When the laminate is bent, the interlaminar failure is consistent with the shear failure, and sliding occurs between the two materials. Therefore, the bending strength of laminates A, B, and C increases with the rise in shear strength. In the case of bending failure, the carbon nanotubes alleviate the damage at the interface in the form of tensile failure, and the carbon nanotubes in the resin will also block the initiation of cracks. Similarly, the strength of the CFRP matrix and ply interface with carbon nanotubes between layers is enhanced, and the bending strength of laminate D is improved. The bending strength of laminate D gets an improvement of about 17.8% over the bending strength of laminate C.

**Figure 13:**
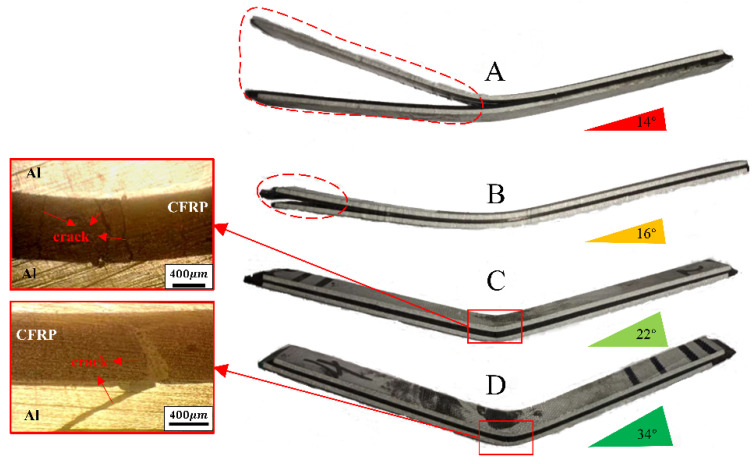
Bending failure forms of four laminates.

The deformation of aluminum plate and CFRP is different under load because of their different material properties. Figure 13 shows the damage caused by bending of four kinds of laminates. The shear strength of laminates A and B is low, obvious delamination occurs between the two materials, and the delamination of laminate A is more serious. When the bending test specimen is made, the direction of the fiber is parallel to the short side of the specimen. Accordingly, the matrix in CFRP will crack first when the laminate is compressed. The bending failure of laminate C is the crack of the CFRP layer in the core, rather than the failure of the Al/CFRP interface. The resistance of laminates to interlayer delamination is stronger when the interfacial strength between the Al/CFRP interface is higher. The form of bending failure of laminate D is the simultaneous cracking of the aluminum plate and CFRP, which is mainly due to the strengthening and toughening effect of the carbon nanotubes added between layers on the matrix. The shear strength of the Al/CFRP interface of laminate D is high, and the fracture of the two materials tends to be the same. The bending angle of the four kinds of laminates is also increasingly becoming larger, which implies that the ability of laminates to resist bending failure is also increasingly becoming better.

### 3.4. Impact Test Results

**Figure 14:**
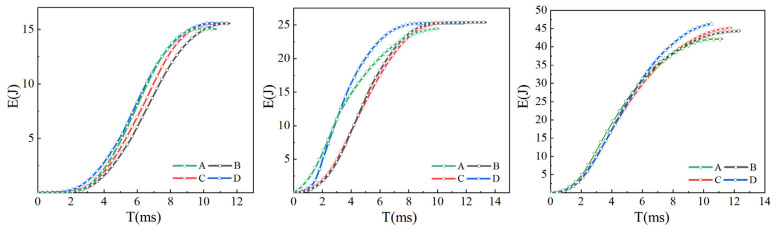
Impact energy absorption curve of laminate.

When the laminate is impacted, delamination occurs at the interface. The occurrence of delamination between layers is less easy and the laminates can consume more impact energy when the interface strength between layers is higher. Figure 14 shows the energy absorption curves of four kinds of laminates under 15 J, 25 J and 45 J impact energy. Under the impact energy of 15 J and 25 J, the energy absorbed by laminate A is less, and the energy absorption of the other three laminates is generally the same. Under 45 J impact energy, the energy absorption of laminates A, B, and C is also increased with the rise in interlaminar shear strength. Carbon nanotubes are added at the interface of the CFRP laminate at the core of laminate D to achieve multi-layer strength and toughness, so that the energy absorbed by laminate D is greater than that of the other laminates. The interlaminar strength is positively related to the impact energy absorption effect of the laminates, and the higher the impact energy is, the more obvious the correlation is. At 45 J impact energy, the matrix interlaminar reinforcement and matrix toughening effect of resin and carbon nanotubes were more obvious.

**Figure 15:**
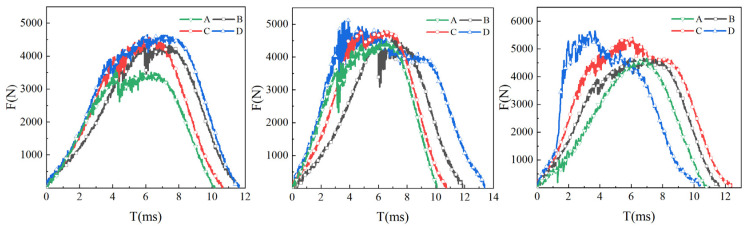
Impact load curve of laminate.

Figure 15 shows the impact load curves of four laminates under 15 J, 25 J and 45 J impact energy. As shown in the figures, the load peak of the impact curve for the four laminates increases successively with the rise in bending strength. The increase in interlaminar shear strength and the strength and toughness between CFRP layers can enable the laminates to bear greater impact load. Delamination is an important form of impact failure in the process of impact. Improving the interlaminar strength can make delamination more difficult, which enhances the impact resistance of laminates.

When the laminate is damaged by impact, the failure form between layers is also similar to that of shear failure. When the laminate is damaged, the interlocking structure of the interlayer interface can resist the mutual movement between the two materials, such as the “W” structure mentioned above. The laminate with resin added to the interface resists the impact damage to the interface through the cracking of resin added and the peeling between aluminum plate and resin added. The damage of carbon nanotubes and two resins at the interface of Al/CFRP can reduce the damage of impact to the interface. Meanwhile, the CFRP interface alleviates the damage caused by impact through the cracking of resin matrix and the fracture of carbon nanotubes. This multi-level structure can improve the energy absorption of the laminate.

**Figure 16:**
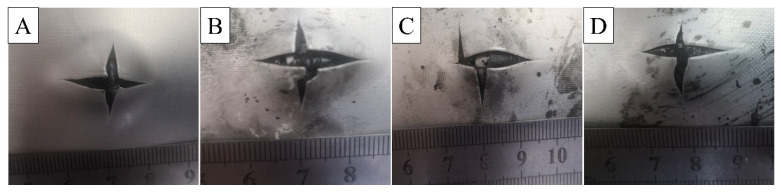
Impact failure morphology of different laminates. (**A**) laminates A. (**B**) laminates B. (**C**) laminates C. (**D**) laminates D.

**Figure 17:**
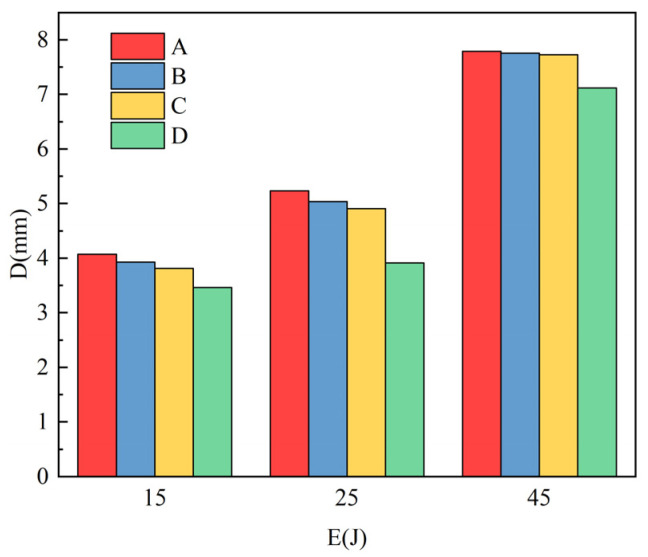
Impact pit depth of different laminates.

Figure 16 shows the failure morphology of four kinds of laminates under 45 J impact energy. After being impacted, the aluminum plate on the back of the laminate produces an “X”-shaped crack and presents a bulging state. The back cracks of laminates C and D are smaller than those of the two other laminates, which indicates that the failure of laminates is related to the interfacial strength of laminates. Figure 17 is a histogram of pit depth after laminate failure. As shown in the figure, the depth of pits on the back of laminates A, B, C, and D gradually becomes smaller under three kinds of energy, which shows that the laminate has increasingly become better able to resist impact failure. The effects of adding resin and carbon nanotubes at the interface on the shear, flexural strength and impact resistance of Al/CFRP/Al laminates are studied. After surface treatment, the laminates have rich interface structures and chemical bonds, so they have higher shear strength and better impact resistance. Carbon nanotubes and epoxy resin were added between Al/CFRP layers to strengthen and toughen the laminates. The impact resistance of laminates with carbon nanotubes added is better than that of laminates without added carbon nanotubes, and the impact resistance of carbon nanotube laminates added between the core CFRP layers will be further improved. When the laminates are damaged by impact, the added carbon nanotubes will be pulled out and broken to mitigate the damage caused by impact.

## 4. Conclusions

The interface is a key part of the laminate. The improvement in interface strength will enhance the performance of the laminate.

- After the surface treatment of the aluminum plate, “W”-type gullies and micropores are produced on the surface of the aluminum plate. When compounding, it will form an interlock structure with resin to improve the interface strength.
- Adding epoxy resin and epoxy resin with carbon nanotubes between Al/CFRP interface layers can improve interlayer shear strength, which can enhance the bending strength and impact resistance of laminates. The bending strength and impact resistance of laminates are positively correlated with the shear strength between layers. The bending strength of laminates can get an improvement of about 104% after adding epoxy resin and epoxy resin with carbon nanotubes between Al/CFRP interface layers.
- After carbon nanotubes are added between CFRP layers, the matrix of adjacent layers will wrap the carbon nanotubes, which will toughen CFRP and further enhance the bending strength and impact resistance of toughened laminates. When the laminates are damaged by shearing, bending, and impacting, the resin between the layers will resist the damage in the form of cracking. The added carbon nanotubes will also resist the damage and the extension of cracks at the interface in the form of pulling out and breaking.

## Figures and Tables

**Table 1:** Four different laminate structures.

Laminate Label	Laminate Structure
A	Al/CFRP/Al
B	Al/e/CFRP/e/Al
C	Al/ec/CFRP/ec/Al
D	Al/ec/CFRPc/ec/Al

## Data Availability

The raw/processed data required to reproduce these findings cannot be shared at this time due to technical or time limitations.
